# Standardized pharmacological management of delirium after on-pump cardiac surgery reduces ICU stay and ventilation in a retrospective pre-post study

**DOI:** 10.1038/s41598-023-30781-y

**Published:** 2023-03-06

**Authors:** Matthias Manfred Deininger, Stefan Schnitzler, Carina Benstoem, Tim-Philipp Simon, Gernot Marx, Despina Panagiotidis, Dmitrij Ziles, Heike Schnoering, Evangelos Karasimos, Thomas Breuer

**Affiliations:** 1 Department of Intensive and Intermediate Care, Medical Faculty, RWTH Aachen University, Pauwelsstr. 30, 52074 Aachen, Germany; 2 Department of Anesthesiology, Medical Faculty, RWTH Aachen University, Aachen, Germany; 3 Department of Cardiovascular Surgery, Medical Faculty, RWTH Aachen University, Aachen, Germany

**Keywords:** Drug delivery, Vascular diseases, Disorders of consciousness, Outcomes research, Disorders of consciousness, Respiratory signs and symptoms, Cardiovascular diseases

## Abstract

Cardiac surgery patients not only undergo a highly invasive procedure but are at risk for a diversity of postoperative complications. Up to 53% of these patients suffer from postoperative delirium (POD). This severe and common adverse event increases mortality and prolonged mechanical ventilation and extends the intensive care unit stay. The objective of this study was to test the hypothesis that standardized pharmacological management of delirium (SPMD) may reduce the length of stay in the intensive care unit (ICU), duration of postoperative mechanical ventilation, and the incidence of postoperative complications such as pneumonia or bloodstream infections in on-pump cardiac surgery ICU patients. In this retrospective, single-center observational cohort study, 247 patients were examined between May 2018 to June 2020, who underwent on-pump cardiac surgery, suffered from POD, and received pharmacological POD treatment. 125 were treated before and 122 after SPMD implementation in the ICU. The primary endpoint was a composite outcome, including the length of ICU stay, postoperative mechanical ventilation time, and ICU survival rate. The secondary endpoints were complications including postoperative pneumonia and bloodstream infections. Although the ICU survival rate was not significantly different between both groups, the length of ICU stay (control group: 23 ± 27 days; SPMD group: 16 ± 16 days; p = 0.024) and the duration of mechanical ventilation were significantly reduced in the SPMD-cohort (control group: 230 ± 395 h; SPMD group: 128 ± 268 h; p = 0.022). Concordantly, the pneumonic risk was reduced after SPMD introduction (control group: 44.0%; SPMD group: 27.9%; p = 0.012) as well as the incidence for bloodstream infections (control group: 19.2%; SPMD group: 6.6%; p = 0.004). Standardized pharmacological management of postoperative delirium in on-pump cardiac surgery ICU patients reduced the length of ICU stay and duration of mechanical ventilation significantly, leading to a decrease in pneumonic complications and bloodstream infections.

## Introduction

Postoperative delirium (POD) is a common adverse event after cardiac surgery. It occurs in up to 53% of these critically ill patients^1–3^. This acute state of confusion, characterized by fluctuating vigilance, inattention, and disorganized thinking^4^, results in prolonged length of hospital stay^3,5^ and increases mortality^6,7^. Delirium in the ICU is associated with increased ventilation duration and longer ICU stay^8^. POD is a multifactorial disturbance that reduces mobility and is also associated with higher hospital readmission rates^9^, lower quality of life, and cognitive failure^2,10^. Contributing factors that influence the incidence of POD include patient age and preexisting comorbidities^11^. Additionally, in cardiac surgery, the use of on-pump cardiopulmonary bypass (CPB) itself, the duration of CPB as well as the intraoperative blood pressure were found to be associated with POD^11–14^.

Early diagnosis of delirium is crucial for optimal treatment. The hypodynamic subtype of POD frequently remains undetected, as it is characterized by a reduction of awareness and muscular activity^15,16^. A systematic screening protocol is required for early POD diagnosis, such as the Confusion Assessment Method for Intensive Care Unit (CAM-ICU). It should be routinely applied to all ICU patients and reevaluated closely^17^.

Treatment options can be distinguished between non-pharmacological and pharmacological therapies. Evidence for individual non-pharmacological^18^ and pharmacological delirium therapy is lacking for ICU patients^19^. As the development of delirium is multifactorial, it seems unlikely that a single intervention or drug can reduce the incidence of delirium^20^. Implementation programs that included treatment bundles and organizational changes for prevention, assessment, and treatment support this hypothesis, as these could improve clinical outcome^21^.

Priority should be given to treating potentially reversible causes, e.g., adequate pain relief and non-pharmacological strategies. According to PADIS guidelines, pharmacological agents should not be administered routinely to delirious patients due to lacking evidence. Nevertheless, patients suffering from severe symptoms secondary to delirium like anxiety, hallucinations, or agitation with self or foreign harmfulness, might benefit from short-term pharmacological therapy^22^. However, to date, there is no standardized, evidence-based approach as to which substance or dosage should be used for the pharmacological treatment of POD.

This retrospective, single-center pre-post study was conducted to test the hypothesis that a standardized pharmacological management of postoperative delirium (SPMD) can reduce the length of stay in the intensive care unit (ICU), duration of postoperative mechanical ventilation, and postoperative complications such as pneumonia or bloodstream infections in on-pump cardiac surgery ICU patients.

## Methods

### Study design and patient population

The study was approved by the Ethics Committee of the Medical Faculty, RWTH Aachen University approval date: January 8th 2021, approval number: EK 509/20. Written patient informed consent was waived by Ethics Committee of the Medical Faculty, RWTH Aachen University because retrospective data were analyzed entirely anonymously. All research procedures were conducted according to the ethical standards of the institutional research committee and the Declaration of Helsinki. This article was created according to the Strengthening the Reporting of Observational Studies in Epidemiology (STROBE) guidelines^23^.

To evaluate the impact of standardization of pharmacological treatment of postoperative delirium, a pre-post comparison was performed between May 2018 and June 2020. Therefore, the control group was studied from May 2018 to April 2019. In May 2019 through June 2019, the SPMD was implemented into clinical routine, and ICU staff were trained in its safe use. After this introductory period, the SPMD group was studied from July 2019 to June 2020.

This study focuses on cardiac surgery patients undergoing CPB. Thus, we included cardiac surgery patients undergoing on-pump coronary artery bypass graft (CABG) surgery, patients with on-pump valve replacement, patients with left ventricular assist device (LAVD) implantation, and patients with supracoronary aortic replacement or a combination of the before mentioned. Exclusion criteria were: patients with isolated thoracic surgery and cardiac surgery without CPB (e.g., off-pump CABG).

### Anesthesia and cardiopulmonary bypass

All surgical procedures, including CABG surgery, valve surgeries, LVAD implants, and surgeries on the ascending aorta/aortic root, were performed under CPB under the same protocol of general anesthesia. A non-pulsatile roller pump was used as a conventional CPB circuit (Stockert s5, Sorin Group Germany, Munich, Germany) with moderate hypothermia (32–34 °C).

Cardiac arrest was induced by antegrade infusion of cold crystalloid cardioplegic solution (CustodiolTM, KoehlerChemie, Alsbach-Haehnlein, Germany) immediately after cross-clamping. Extracorporeal circulation was performed with a non-pulsatile pump flow of 2.2 L min^−1^ m^−2^. Patients were weaned from CPB after rewarming and aortic declamping resulting in sufficient reperfusion. Blood transfusion was applied to maintain hemoglobin levels above 7.5 mg/dL, and hemodilution was limited by maintaining hematocrit levels above 21%. The vasopressor noradrenaline and, in inotropic doses, epinephrine were applied when required during and after the operation. Immediately after the procedure, all patients were transferred to the ICU. Thrombosis prophylaxis with unfractionated heparin was initiated as soon as possible after cardiac surgery. If indicated, therapeutic anticoagulation was initialized and maintained during the ICU stay for patients who had no active bleeding. The patients developing prolonged postoperative atrial fibrillation were anticoagulated with intravenous PTT-guided therapeutic doses of unfractionated heparin.

### Diagnosis of delirium

The Richmond Agitation Sedation Scale (RASS) was performed at least twice daily to assess the mental status and detect acute onsets and orientation fluctuations in all patients^24^. Furthermore, the CAM-ICU was applied once during every 12-h shift^25^. Postoperative delirium was diagnosed, if there was at least one positive CAM-ICU test in combination with further clinical signs supporting the POD diagnosis.

### Standardized pharmacological management of delirium

**Figure 1:**
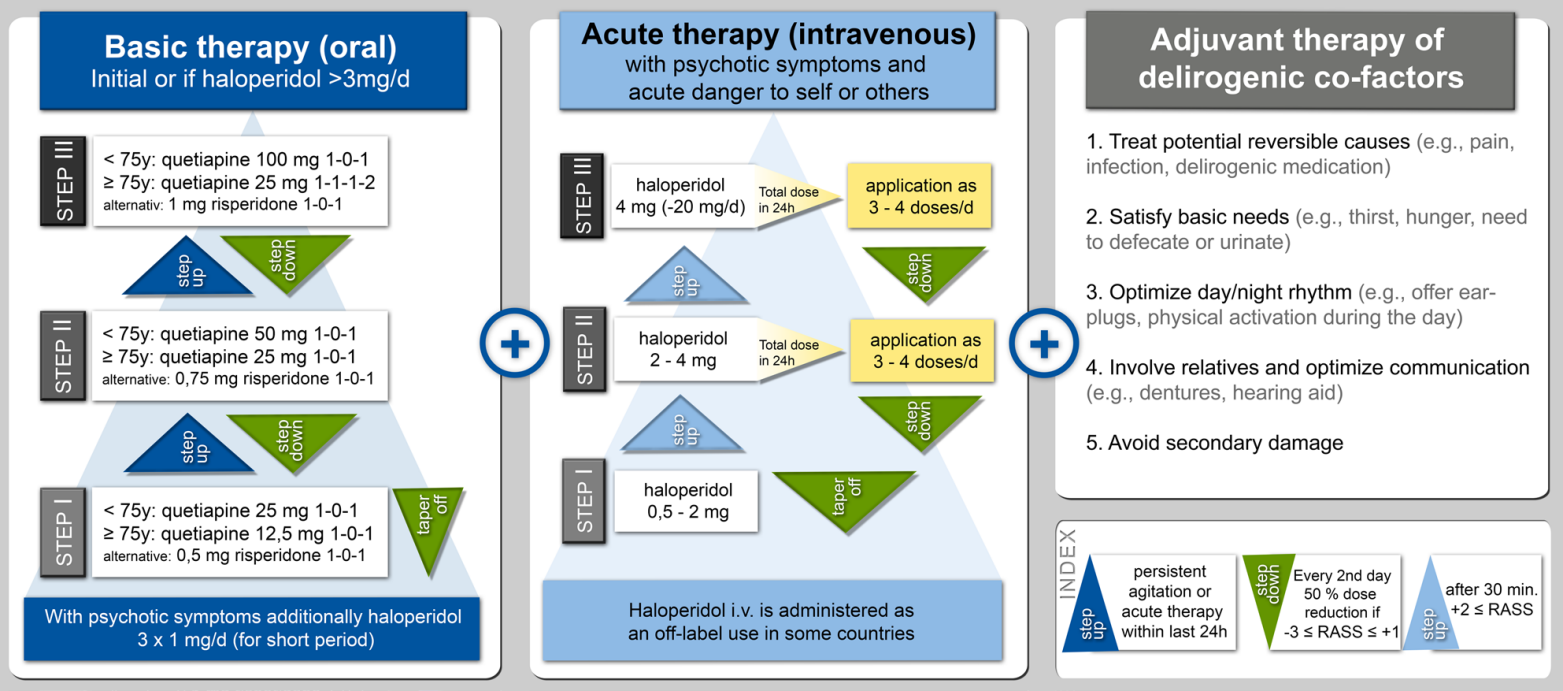
Standardized pharmacological management protocol for postoperative delirium. 3-step standardized age-related pharmacological management protocol for postoperative delirium. Initial treatment is started with step 1 and adjusted by step-up or -down depending on the patient's Richmond Agitation Sedation Scale (RASS). The step-down approach is characterized by halving the dose every other day until medication can be stopped or until symptoms are again exacerbating. The latter could lead to a reversion into the step-up approach increasing the overall dosage. This protocol is continued until delirium is adequately treated and tapered off when delirium ends.

In May 2019, we introduced the standardized pharmacological management of delirium (SPMD) as a novel standardized operating procedure in our cardiac surgical ICU. The non-pharmacological POD treatment strategies, including treatment of potentially reversible causes (e.g., pain), satisfying basic needs (e.g., thirst, hunger), optimizing circadian rhythm, physical activation during the day, and involvement of relatives, were provided continuously in our ICU throughout the observation period. The SPMD followed the goal to complement these existing non-pharmacological strategies with standardized indication, dosage, and tapering for pharmacological treatment of POD (Fig. 1). Therefore, oral anti-delirium medication was used as the baseline medication. Acute endangering behavior to self or others was additionally treated with intravenously administered haloperidol^26^. An essential element of our standardized pharmacological POD therapy was the age-related, gradual escalation and tapering according to RASS.

### Data collection

The demographic pre-, intra- and postoperative data of all patients (125 patients in the control group and 122 in the SPMD group) were collected in a Microsoft Excel spreadsheet (Microsoft 365 MSO, Version 2112, Redmond, USA) from medical files, intraoperative anesthesia, and perfusion charts. Variables were collected as follows: (i) Preoperative characteristics: age, sex, medical history including arterial hypertension, diabetes, stroke/transient ischemic attack (TIA), psychiatric disorders (e.g., depression, schizophrenia, dementia), chronic kidney disease, and the pulmonary disorders asthma and chronic obstructive pulmonary disease (COPD). (ii) Perioperative characteristics: Surgical procedure, American Society Anesthesiology (ASA) physical status classification system, simplified acute physiology score (SAPS II) on ICU admission, the classification as emergency surgery, times of surgery, CPB and aortic clamping, as well as the number of red blood cell (RBC) and fresh frozen plasma (FFP) transfusions administered intraoperatively.

### Study endpoints

The primary endpoints included the duration of postoperative mechanical ventilation, ICU stay, and ICU survival rate of patients with delirium after different cardiac surgery procedures with CPB. The secondary endpoints were the incidences of (i) re-intubation, (ii) postoperative pneumonia, (iii) bloodstream infections, and (iv) other complications like sternal wound infection, urinary tract infection, and acute kidney injury.

### Subgroup analyses

Subgroup analyses were performed based on age, SAPS II, and CPB time. To our knowledge, there are no established cutoff values or ranges related to outcome for the above parameters in cardiac surgery ICU patients. Therefore, we used the median across both study groups of each parameter as a cutoff, which allowed equivalent subgroup sizes. Given the total number of patients, only two groups were formed per analysis.

### Statistical analysis

We formally tested for normality and homogeneity of variance of the residuals by applying Shapiro–Wilk’s test and Levene’s test, respectively, with α = 0.05. Comparisons between the groups for the variables “duration of mechanical ventilation” and “ICU length of stay” were made by a non-parametric Mann–Whitney-*U*-test. Unpaired Student's *t* test was used for parametric distributed continuous samples. All other secondary endpoints were tested using Fisher’s exact test. When appropriate, data are shown as mean ± SD, median (range), or absolute numbers. All statistical tests are two-tailed. Significance was defined as p < 0.05. Statistical analysis was performed using Prism 8.0 (GraphPad Software Inc., San Diego, CA, USA) and SPSS version 28 (IBM Corp., Armonk, NY).

## Results

### Study population

**Figure 2:**
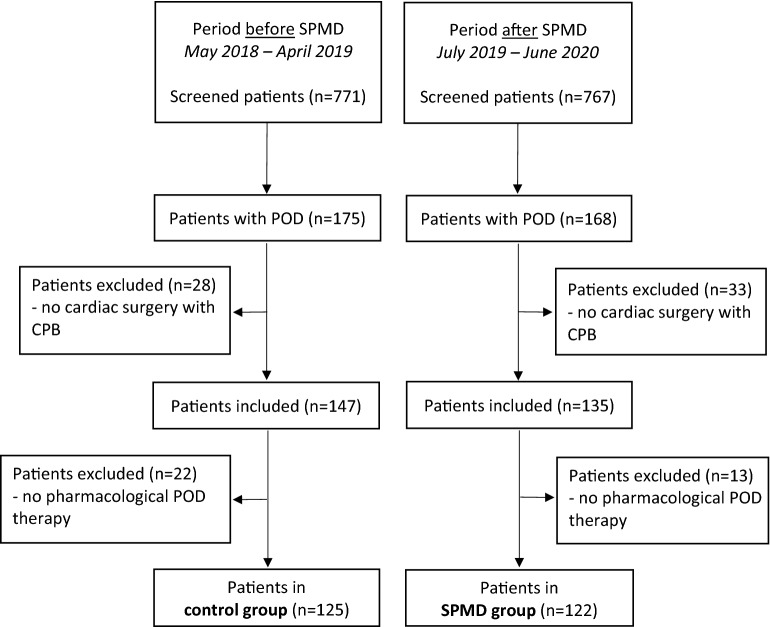
Study population flow chart. *CPB* cardiopulmonary bypass, *POD* postoperative delirium, *SPMD* standardized pharmacological management of delirium.

The data of 1538 patients admitted to the cardiac surgery intensive care unit at the Department of Intensive and Intermediate Care, RWTH Aachen University, between May 2018 and June 2020 were retrospectively collected (Fig. 2). 771 patients before (control cohort) and 767 after SPMD implementation (SPMD cohort) were screened. The control cohort was set from May 2018 to April 2019. After the SPMD introduction, the SPMD cohort was studied from July 2019 to June 2020.

In total, 343 (22.3%) were diagnosed with POD. No significant difference could be shown for the incidence of POD between the cohorts before and after SPMD introduction [control cohort: 175 of 771 (22.7%); SPMD cohort: 168 of 767 (21.9%), p = 0.714]. After excluding patients not meeting the previously defined inclusion criteria, 282 POD patients undergoing cardiac surgery with CPB remained (147 patients before and 135 patients after implementing the SPMD). No significant difference in the likelihood of POD was found between the two study groups (p = 0.469). All patients with sole non-pharmacological POD therapy were excluded from subsequent analyses to evaluate the impact of standardized pharmacological management. This resulted in 125 patients (85.0%) in the control group and 122 patients (90.3%) in the SPMD group. No difference in the frequency of pharmacological POD therapy was found before and after SPMD implementation (p = 0.207).

Patient characteristics, including medical history and the classification of the surgical procedure, are shown in Table 1. There were no significant differences in patient characteristics, surgical procedures, perioperative treatment and times, expect the group of double coronary artery bypass graft surgeries [double CABG, SPMD group 27 of 122 patients (22.1%); control group 14 of 125 patients (11.2%); p = 0.026]. Concordant, the number of triple CABG in the SPMD group was—although statistically non-significant—reduced (SPMD group: 35 of 122 patients (28.7%); control group 50 of 125 patients (40.0%); p = 0.081).

**Table 1:** Demographic and surgery data.

Variable	Group comparison		
	Control (n = 125)	SPMD (n = 122)	*p*-value
Patient characteristics and medical history [*n* (%)]			
Age in years [median (range)]	73 (38–84)	73 (46–85)	0.269
Sex (male)	100 (80.0%)	89 (73.0%)	0.230
Arterial hypertension	101 (80.8%)	93 (76.2%)	0.439
Diabetes	39 (31.2%)	37 (30.3%)	0.891
Stroke/TIA	18 (14.4%)	11 (9.0%)	0.236
Psychiatric disorder	10 (8.0%)	12 (9.8%)	0.660
Chronic kidney disease	12 (9.6%)	16 (13.1%)	0.426
Asthma	2 (1.6%)	3 (2.5%)	0.681
COPD	14 (11.2%)	10 (8.2%)	0.521
Surgical procedure [*n* (%)]			
Single CABG	7 (5.6%)	6 (4.9%)	1.000
Double CABG	14 (11.2%)	27 (22.1%)	**0.026**
Triple CABG	50 (40.0%)	35 (28.7%)	0.081
Quadruple CABG	16 (12.8%)	19 (15.6%)	0.587
Quintuple CABG	1 (0.8%)	0 (0%)	1.000
With valve repair	17 (13.6%)	18 (14.8%)	0.856
With LVAD	2 (1.6%)	0 (0%)	0.498
With aortic repair	3 (2.4%)	1 (0.8%)	0.622
Ascending aorta/aortic root repair	13 (10.4%)	20 (16.4%)	0.193
With valve repair	1 (0.8%)	5 (4.1%)	0.117
Singular valve repair	22 (17.6%)	15 (12.3%)	0.286
Singular LVAD-implantation	2 (1.6%)	0 (0%)	0.498
Anesthesiology [mean ± SD]			
ASA-Score	3.4 ± 0.6	3.3 ± 0.6	0.262
SAPS II Score	33 ± 9	34 ± 8	0.330
Emergency surgery [*n* (%)]	21 (16.8%)	18 (14.8%)	0.728
Surgery time in minutes	278 ± 76	260 ± 106	0.140
CPB-time in minutes	135 ± 54	138 ± 58	0.631
Aortic clamping time in minutes	79 ± 36^A^	81 ± 36	0.799
RBC transfusion intraoperative	2.3 ± 2.1	2.7 ± 2.7	0.439
FFP transfusion intraoperative	0.6 ± 1.6	0.8 ± 2.4	0.758

Data are presented as mean ± SD, median (range), or absolute numbers (with percentage of the group [%]). ^A^Two missing values (n = 123) due to incomplete documentation.

*ASA* American society anesthesiology, *CABG* coronary artery bypass graft, *COPD* chronic obstructive pulmonary disease, *CPB* cardiopulmonary bypass, *FFP* fresh frozen plasma, *LVAD* left ventricular assist device, *SAPS II* simplified acute physiology score, *SPMD* standardized pharmacological management of delirium, *RBC* red blood cell, *TIA* transient ischemic attack.

Significant values are in bold.

### Reduction of haloperidol usage after SPMD implementation

**Figure 3:**
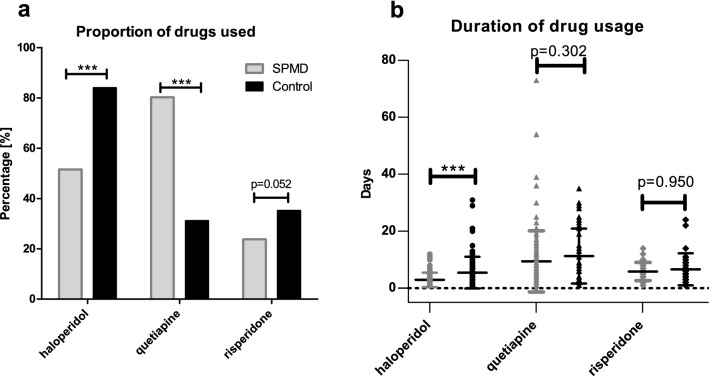
Comparison of drug usage for delirium therapy between control and SPMD group. Percentage of post cardiac surgery ICU patients who received the corresponding drug for delirium therapy is shown in the figure. In some patients, more than one substance was used. A differentiation is made between the group treated according to standardized pharmacological management for postoperative delirium (SPMD) and control group before the SPMD introduction. In the control group 125 patients and in the SPMD group 122 patients were studied; ***p < 0.001.

To evaluate the impact of SPMD implementation on drug usage for pharmacological management of delirium the three drugs included in the SPMD protocol were compared before and after SPMD implementation as shown in Fig. 3a. After introducing the SPMD, haloperidol usage was significantly reduced from 84 to 51.6% (p < 0.001), whereas quetiapine usage was significantly increased from every third patient to 80.3% of the patients (p < 0.001). The usage of risperidone did not change significant (control: 35.2%; SPMD: 23.8%; p = 0.052). In terms of application duration a significant reduction could be seen for haloperidol (control group: 5.5 ± 5.5 days; SPMD group: 3.0 ± 2.5 days; p < 0.001) after SPMD implementation, whereas for quetiapine (control group: 11.3 ± 9.7 days; SPMD group: 9.4 ± 10.7 days; p = 0.302) and risperidone (control group: 8.9 ± 15.8 days; SPMD group: 5.9 ± 3.2 days; p = 0.950) there was no significant change (Fig. 3b).

### Reduced length of ICU stay and ventilator time after SPMD implementation

**Figure 4:**
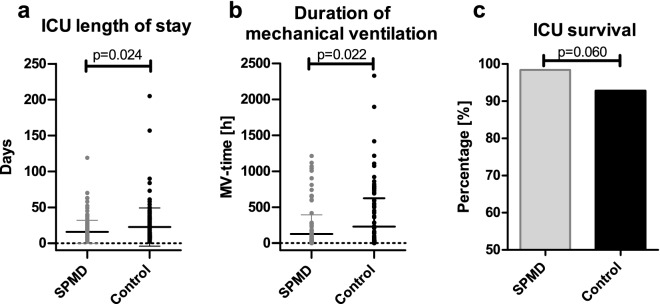
Duration of ICU stay, mechanical ventilation and ICU-survival rate. The length of the intensive care unit stay in days is depicted in section (**a**), comparing the effect of the standardized pharmacological management for postoperative delirium (SPMD group) with the control group before the SPMD introduction. The duration of mechanical ventilation (MV-time) is illustrated in section (**b**), and section (**c**) shows the relative ICU-survival rate for the SPMD (120 of 122 patients) and the control group (116 of 125 patients). The MV-time in hours and the ICU length in days are plotted separately for every patient. Mean ± SD is shown as horizontal lines in sections (**a**) and (**b**).

After implementing the SPMD in the treatment of on-pump cardiac surgery patients, we observed a significant reduction in the length of ICU stay (p = 0.024, Fig. 4a). In the SPMD group, the patients spent on average 16 ± 16 days in the ICU, whereas in the control group the length of ICU stay was 23 ± 27 days. The average ventilator time (duration of mechanical ventilation) was reduced significantly from 230 ± 395 h in the control group to 128 ± 268 h in the SPMD group (p = 0.022, Fig. 4b). ICU-survival rate was 98.4% in the SPMD group (120 of 122 patients) and 92.8% in the control group (116 of 125 patients, p = 0.060, Fig. 4c).

### Reduction of pneumonia and bloodstream infection rate

**Figure 5:**
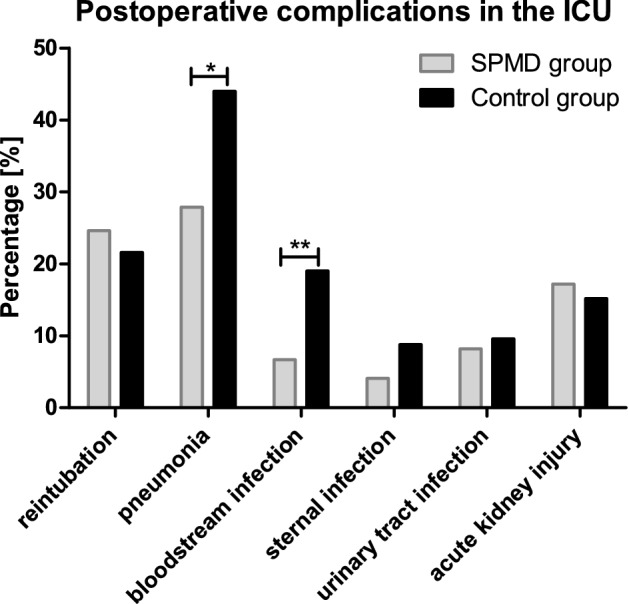
Postoperative infections or organ dysfunction. Complications of on-pump cardiac surgery ICU patients with POD are plotted as percentages of the respective group, contrasting standardized pharmacological management for postoperative delirium (SPMD), and control group before the SPMD introduction. In the control group 125 patients and in the SPMD group 122 patients were studied; *p = 0.012; **p = 0.004.

As secondary endpoints the incidence of complications in the ICU were assessed (Fig. 5). The rate of reintubation was not statistically different before and after implementing the SPMD on the cardiac surgical ICU with 21.6% in the control group and 24.6% in the SPMD group (p = 0.651). The incidence pneumonia was significantly reduced after SPMD introduction (control group: 44.0%; SPMD group: 27.9%; p = 0.012). Also, the incidence of blood stream infection was reduced significantly from 19.2% (24 of 125 patients) in the control group to 6.6% in the SPMD group (9 of 122 patients; p = 0.004). For the sternal wound infection (control group: 8.8%; SPMD group: 4.1%; p = 0.196), urinary tract infection (control group: 9.6%; SPMD group: 8.2%; p = 0.824) and acute kidney injury (control group: 15.2%; SPMD group: 17.2%; p = 0.731) the incidences did not differ significantly between the two groups.

### Effect of SPMD differs by age and disease severity

To examine whether the outcome differences seen after implementation of the SPMD were primarily attributable to specific patient groups, we performed subgroup analyses. On the one hand, we investigated the influence of patient age and on the other hand, the significance of disease severity on ICU admission, represented by the SAPS II score and CPB-time. The latter can also be considered as a surrogate parameter for the complexity of the cardiac surgery. Using the median patient age of 73 years as a cutoff, there was a significant difference between the control and SPMD groups for patients 73 years and younger in terms of ICU length of stay (control group: 24 ± 29 days; SPMD group: 15 ± 17 days; p = 0.038), duration of mechanical ventilation (control group: 292 ± 438 h; SPMD group: 126 ± 282 h; p = 0.003), bloodstream infections (control group: 23.8%; SPMD group: 3.0%; p < 0.001), and pneumonia (control group: 47.6%; SPMD group: 25.8%; p = 0.011), whereas no significant differences were found for patients older than 73 years between SPMD and control group (Supplements, Fig. S1). Analogous to age, median SAPS II score of 33 or less was found to be associated with significant differences between the SPMD and control groups in terms of ICU length of stay (control group: 22 ± 28 days; SPMD group: 14 ± 13 days; p = 0.027), duration of mechanical ventilation (control group: 195 ± 376 h; SPMD group: 87 ± 207 h; p = 0.044), bloodstream infections (control group: 18.1%; SPMD group: 5.1%; p = 0.031), and pneumonia (control group: 41.7%; SPMD group: 22.0%; p = 0.024). However, for patients with SAPS II above 33 no significant group differences could be shown (Supplements, Fig. S2).

The subgroup analysis with median of CPB-time (128 min) as cutoff showed for duration of mechanical ventilation a significant difference between the control and the SPMD group (control group: 325 ± 493 h; SPMD group: 185 ± 330 h; p = 0.037) as well as for blood stream infections (control group: 19.7%; SPMD group: 6.5%; p = 0.034) when CPB-time was longer than 128 min. A significantly reduced pneumonic incidence was shown in the SPMD in comparison to the control group (control group: 37.5%; SPMD group: 18.3%; p = 0.027) when CPB-time was maximal 128 min (Fig. S3).

## Discussion

National and international guidelines, like PADIS, mention various drugs for the pharmacological therapy of delirium^22,27,28^. Nevertheless, no effective pharmacological treatment method for postoperative delirium has been identified so far^19^. Thus, a uniform recommendation for both substance and dosage are missing due to lack of evidence. Haloperidol and atypical antipsychotics are, according to the recent international survey *SAMDS-ICU*, used by two of three ICU physicians for pharmacological POD treatment^29^. Studies comparing these substance classes could not show significant differences^30–32^. Considering that pharmacological treatment of POD is continued after ICU discharge in about 20%, there may be increased side effects and adverse outcomes^33–36^. Thus, besides the aspect of rational, limited use, especially the short-term aspect of pharmacological management for POD, is relevant. In 2019, D’Angelo and colleagues showed that the usage of pharmacological POD tapering bundles could significantly reduce the number of antipsychotic continuations after ICU- and hospital-discharge^37^.

Therefore, the SPMD introduced in our ICU aimed to standardize and rationalize the use, duration, dosage, and tapering off the drugs used for pharmacological POD treatment in a RASS-dependent manner. Moreover, SPMD limited the use of haloperidol to delirium patients with hallucinatory symptoms and danger to self or others. As the data of this retrospective single-center study showed, the implementation of the SPMD resulted in a significant reduction in overall haloperidol use and duration, whereas quetiapine usage, as basic SPMD treatment drug (Fig. 1), increased significantly (Fig. 3). The duration of treatment per patient, however, did not change significantly for quetiapine.

According to the ABCDEF-approach for delirium, the optimal level of vigilance plays an essential role, especially with regards to the feasibility of non-pharmacological delirium measures, which are crucial for the best possible treatment^38^. It seems plausible that through the RASS-guided daily dose adjustment and rational usage of pharmacological POD treatment in SPMD an improvement in clinical management could be achieved without the risk of prolonged treatment or under- as well as overdose. Focusing on the endpoints of this study, the implementation of SPMD resulted in a significant reduction of the duration of mandatory ventilation and—concordant with that—also a decline in pneumonic complications and bloodstream infections (Figs. 4, 5). A meta-analysis showed that the risk of pneumonia increases with antipsychotic treatment, especially in older age^39^. This might be due to increased sedation and highlights the importance of minimizing duration and dosage of pharmacologic POD therapy. It seems therefore plausible, that the SPMD implementation reduced the pneumonia rate in POD patients.

Based on a meta-analysis, POD-bundles combining pain, agitation, and delirium management strategies were more likely to reduce mortality and length of ICU stay than single intervention strategies^21,40^. A prospective multicenter pre-post intervention study could show that programs implementing PADIS guidelines could improve bundle adherence and reduce the duration of delirium but did not influence mortality, length of stay, or mechanical ventilation^41^. The bundle of non-pharmacological and standardized pharmacological POD therapy used in this study showed a significant reduction in the ICU length of stay compared to the control period (Fig. 4a). This is most likely due to the decrease in complications achieved by the rational and short-term administration period of pharmacological POD therapy rather than the specific drugs used. Although statistics showed no significant difference between the groups for ICU mortality, in the SPMD group 2 out of 122 (1.6%) and in the control group 9 out of 125 (7.2%) patients did not survive ICU (p = 0.060).

Based on the subgroup analysis, it can be hypothesized that younger POD patients (≤ 73 years) as well as ICU patients with less severe illness (SAPS II ≤ 33) and patients suffering from longer cardiopulmonary bypass time (> 128 min) are more likely to benefit from SPMD therapy. Considering that morbidity statistically increases with age, it seems plausible that elderly have a fundamentally higher rate of ICU complications and longer ventilation and intensive care stays^42^. Correspondingly, the same applies to POD patients with a higher degree of disease severity on admission^43^. Given the endpoints of this study, it therefore seems plausible that there was no significant effect for SPMD in the older, severely ill patient subpopulation studied, but as the Figs. S1–S3 in the supplement show, a predominantly positive trend could be demonstrated here after SPMD implementation as well.

We note that this study has several limitations. Due to the retrospective nature of the study design, it cannot be fully excluded that differences in the intensive care treatment of patients over the observation period may have influenced the results of this study. Non-pharmacological POD treatment, nevertheless, was unchanged throughout the observation period. The percentage of patients with POD receiving pharmacological delirium treatment is higher than the average reported by Boncyk et al. with about 45%^34^; however, the unique character and increased morbidity of cardiac surgery patients should be considered. The explorative, retrospective nature of our study results in a limited database and the inability to track the total dosage and daily administration frequency of the drugs administered for POD as well as the duration of the POD. Moreover, the success of standardization depends on the practicability and compliance to SPMD adherence of the ICU staff. The numbers of patients in the subgroup analyses were relatively small, and differential confounding exclusion could not be performed, so that these post analyses can primarily be considered as hypothesis generating for further studies which should investigate the potential beneficial effects of SPMD within prospective, randomized controlled trials.

## Conclusions

Standardized pharmacological management of postoperative delirium in on-pump cardiac surgery patients significantly reduced ICU length of stay, duration of mechanical ventilation, the incidence of pneumonia and bloodstream infections. It, therefore, may provide a practical improvement in POD management for these patients.

## Supplementary Information

Supplementary Figures.

## Data Availability

The datasets used and/or analyzed during the current study are available from the corresponding author on reasonable request.
