# Supplementary material for: Standardized pharmacological management of delirium after on-pump cardiac surgery reduces ICU stay and ventilation in a retrospective pre-post study
Source: Sci Rep. 2023 Mar 6;13:3741. doi: 10.1038/s41598-023-30781-y (PMC9988974; doi:10.1038/s41598-023-30781-y)
Supplement: Supplementary file 1 — Supplementary Figures. [file 41598_2023_30781_MOESM1_ESM.docx]

**Supplementary material**

**Standardized pharmacological management of delirium after on-pump cardiac surgery reduces ICU stay and ventilation in a retrospective pre-post study**

Matthias Manfred Deininger^1*^, Stefan Schnitzler^1^, Carina Benstoem^1^, Tim‑Philipp Simon^1^,

Gernot Marx^1^, Despina Panagiotidis^2^, Dmitrij Ziles^2^, Heike Schnoering^3^, Evangelos Karasimos^1^ & Thomas Breuer^1^

Affiliations:

^1^ Department of Intensive and Intermediate Care, Medical Faculty, RWTH Aachen University, Aachen, Germany

^2^ Department of Anesthesiology, Medical Faculty, RWTH Aachen University, Aachen, Germany

^3^ Department of Cardiovascular Surgery, Medical Faculty, RWTH Aachen University, Aachen, Germany

* corresponding author: mdeininger@ukaachen.de

**Figure S1. Age-based subgroup analysis**

The role of patient age on endpoints is illustrated in this figure. Therefore, the median age

(73 years) was used as subgroup cutoff. The standardized pharmacological management for postoperative delirium group (SPMD group) and the control group are compared in terms of the length of the intensive care unit (ICU) stay in section **a**, in terms of duration of mechanical ventilation (MV-time) in section **b**, section **c** shows the relative ICU-survival rate for both groups and section **d** depicts the ICU complications. The MV-time in hours and the ICU length in days are plotted separately for every patient. Mean ± SD is shown as horizontal lines in sections a and b.

**Figure S2. SAPS II-based subgroup analysis**

The relevance of simplified acute physiology score (SAPS II) on the endpoints is illustrated in this graph. As cutoff the median SAPS II of 33 was used. The standardized pharmacological management for postoperative delirium group (SPMD group) and the control group are compared for both subgroups with a SAPS II Score of at least 34 or 33 and below. The length of the intensive care unit (ICU) stay is compared in section **a**, the duration of mechanical ventilation (MV-time) in section **b**, the relative ICU-survival rate in section **c** and section **d** compares ICU complications between the groups. The MV-time in hours and the ICU length in days are plotted separately for every patient. Mean ± SD is shown as horizontal lines in sections a and b.

**Figure S3. CPB-time-based subgroup analysis**

The influence of cardiopulmonary bypass (CPB) surgery time on the endpoints is shown in this figure. As cutoff the median CPB-time of 128 minutes was used. The standardized pharmacological management for postoperative delirium group (SPMD group) and the control group are compared for the subgroup up to and over 128 minutes CPB-time. The length of the intensive care unit (ICU) stay is compared in section **a**, the duration of mechanical ventilation (MV-time) in section **b**, the relative ICU-survival rate in section **c** and section **d** compares ICU complications between the groups. The MV-time in hours and the ICU length in days are plotted separately for every patient. Mean ± SD is shown as horizontal lines in sections a and b.
